# Risk signal assessment of Tdap vaccine use alone by pregnant women: An updated pharmacoepidemiological study

**DOI:** 10.3389/fcimb.2025.1689560

**Published:** 2026-01-27

**Authors:** Rujuan Fan, Zhijuan Zhu, Xiangyu Li, Lingjing Yuan

**Affiliations:** 1 Shaoxing Second Hospital, Shaoxing, Zhejiang, China; 2 Department of Pharmacy, Shaoxing Keqiao Women & Children's Hospital, Shaoxing, Zhejiang,, China

**Keywords:** adverse events, pharmacoepidemiological, pregnancy, surveillance, Tdap, vaccine safety

## Abstract

**Background:**

Despite global recommendations for maternal tetanus-diphtheria-acellular pertussis (Tdap) vaccination to protect neonates, safety concerns persist regarding pregnancy-specific adverse events (AEs). We conducted a 20-year analysis of U.S. Vaccine Adverse Event Reporting System (VAERS) data (2005–2024) to characterize Tdap-associated AEs during pregnancy, with emphasis on understudied outcomes like chorioamnionitis.

**Methods:**

We analyzed 870 pregnancy-related reports (128 serious) from 20,358 Tdap submissions using disproportionality analysis (Reporting Odds Ratio, ROR). Cases were identified via MedDRA-coded terms and free-text mining. Outcomes were stratified by System Organ Class (SOC), trimester, and AE seriousness. Novel signals were evaluated against product labeling and prior studies.

**Results:**

71.9% of AEs occurred within 30 days post-vaccination. No AEs of maternal death were found. Strong safety signals stillbirth (ROR 285.77, IC 8.01), preterm delivery (ROR 196.8, IC 7.51), and fetal death (ROR 140.83, IC 7.06) were found via disproportionality from a passive surveillance system, which required further validation in active surveillance studies. Pregnancy-specific AEs represented 22.3% of significant PTs, including 6 chorioamnionitis cases with most cases having documented obstetric risk factors. Novel signals fetal hypokinesia and urinary tract infection emerged as unlabeled risks.

**Conclusion:**

This analysis affirms Tdap’s favorable safety profile while detecting statistically significant signals that merit additional research. The low absolute risk supports continued maternal immunization, but warrants enhanced monitoring in high-risk pregnancies. The findings highlight the importance of ongoing post-marketing surveillance for evaluating vaccine safety in pregnant populations and contribute to evidence-based decision-making in clinical practice and public health policy.

## 1 Introduction

Pertussis, an acute respiratory infectious disease caused by *Bordetella pertussis*, typically manifests as mild febrile illness in uncomplicated cases. Notably, co-infections with other respiratory pathogens frequently exacerbate clinical outcomes, potentially progressing to severe complications including acute bronchitis, viral pneumonia, infant respiratory distress syndrome, encephalopathy, and fatal cases (Ayala et al., 2011). Despite childhood immunization programs reducing mortality, waning immunity and recent resurgences (epidemiologic surveillance data from 2023-2024) necessitate re-evaluation of maternal Tdap strategies (Klein et al., 2012). Most severe cases and fatalities occur in infants under 3 months of age who have not completed their primary immunization series (Straney et al., 2016). Globally, pertussis remains a severe threat to infants, as evidenced by the WHO’s estimate of 160,000 under-five deaths in 2014 (Yeung et al., 2017). China is experiencing a substantial and epidemiologically significant pertussis outbreak. Between June 2023 and May 2024, a total of 25 (mortality of 0.026%) in infants (mostly under 3 months) deaths were reported (Yahong et al., 2024).

Vaccination is widely recognized as the most effective method for preventing infectious diseases (Li et al., 2024a, 2025). Although adolescents and adults do not exhibit the highest morbidity and mortality rates for whooping cough, they serve as the primary carriers of the disease. To reduce disease burden, some countries implement strategies such as pertussis vaccination for pediatric healthcare workers, postpartum women, and close contacts of infants under 12 months, along with adolescent booster immunization. However, these approaches fail to provide substantial protective effects for infants under 6 months (Forsyth et al., 2015). Crucially, maternal immunization with pertussis-containing vaccines during pregnancy achieves dual protection: direct protection of gravidae and transplacental antibody transfer to neonates (Gall et al., 2011). In October 2011, the Advisory Committee on Immunization Practices (ACIP) of the U.S. Centers for Disease Control and Prevention recommended routine administration of tetanus toxoid, reduced diphtheria toxoid, and acellular pertussis vaccine (Tdap) during pregnancy weeks 27–36 for individuals without prior pertussis vaccination. By 2012, this recommendation expanded to include all pregnant persons regardless of vaccination history (Centers for Disease Control and Prevention (CDC), 2013; Committee opinion no. 718: update on immunization and pregnancy: tetanus, diphtheria, and pertussis vaccination, 2017). Like others, some countries have implemented maternal Tdap vaccination strategies to control the ongoing pertussis pandemic and directly protect newborns during their most vulnerable early life period. However, current pharmacovigilance data remain inconclusive regarding Tdap safety in pregnancy. The most frequently reported potential AEs include injection site reactions (pain, erythema/redness, and/or induration/swelling) according to previous studies (Clark and Johnson, 2023). In addition, existing systematic reviews constrained by methodological limitations in observational studies: inadequate power to detect rare adverse drug reactions (ADRs), incomplete long-term follow-up, and real-world implementation variances (Nasser et al., 2020; Vygen-Bonnet et al., 2020; Munoz et al., 2014).

To address these persistent evidence gaps, this study leverages data from the U.S. Vaccine Adverse Event Reporting System (VAERS). As a national passive surveillance system, VAERS serves as a crucial post-licensure safety monitoring tool, collecting voluntarily submitted reports of suspected adverse events following immunization. Critically, VAERS has become a vital resource for data mining, statistical analysis, and text analysis in vaccine safety surveillance (Li et al., 2024c; Guo et al., 2022). Majority of Adverse Drug Events (ADEs) are preventable, and lessons learned from previous ADEs provide guidance for the prevention of future ADEs (Li et al., 2024d). In this study, we evaluated adverse event reports following Tdap vaccination reported to the VAERS from June 2005 through December 2024 involving pregnant individuals or their infants. This assessment aims to provide new evidence for updating the safety profile of Tdap vaccination during pregnancy and offers novel directions for post-marketing vaccine safety surveillance. This pharmacovigilance study employed VAERS data to investigate potential Tdap vaccine safety signals through three analytical phases: (i) comprehensive case identification of reported AEs without data completeness exclusions; (ii) temporal and demographic characterization of AE patterns, including onset timing post-vaccination and age-specific distributions; and (iii) disproportionality assessment to detect statistically significant AE-vaccine associations.

## 2 Materials and methods

### 2.1 Data source

Established in 1990 under the National Childhood Vaccine Injury Act (1986), the VAERS serves as a passive surveillance platform for monitoring post-immunization AEs (Tatang et al., 2022). This voluntary reporting mechanism accepts submissions from multiple sources including healthcare providers, pharmaceutical manufacturers, vaccine recipients, and their families. The standardized reporting forms document patient demographics, vaccine details, adverse event descriptions, and reporter information, with all entries systematically coded using Preferred Terms (PTs) of the Medical Dictionary for Regulatory Activities (MedDRA) for consistent data analysis. De-identified primary surveillance data are accessible via two public platforms: the VAERS online portal and CDC’s WONDER epidemiological research interface. This global reporting system accepts submissions from US and non-US sources. The classification of serious adverse events (SAEs) in this study strictly adhered to the Code of Federal Regulations criteria, wherein VAERS reports were categorized as serious upon meeting any of the following clinical endpoints: (1) fatal outcome; (2) hospitalization or prolonged hospital stay; (3) life-threatening condition; or (4) persistent/significant disability/incapacity (Li et al., 2024b). Notably, hospital admissions exclusively for uncomplicated obstetric delivery were systematically excluded from SAE classification to enhance the specificity of safety signal detection. As per regulatory requirements, comprehensive documentation of both medical history and immunization records was mandated for all case reports submitted to VAERS, irrespective of their severity classification.

### 2.2 Data processing

A flow diagram of data extraction and mining is shown in **Supplementary Figure S1**. A systematic query of the VAERS database was performed to identify reports involving pregnant individuals administered Tdap vaccine alone within the United States from June 10, 2005, through December 31, 2024. The search methodology followed established protocols (Moro et al., 2016), incorporating: 1. MedDRA 27.0-coded terms within the System Organ Classes of Pregnancy, Puerperium, and Perinatal Conditions (10036585) and Congenital, Familial and Genetic Disorders (10010331); 2. Specific PTs (Drug Exposure during Pregnancy, Maternal Exposure during Pregnancy, Exposure during Pregnancy); 3. and A free-text search for the string “preg” within narrative fields. Additionally, we omitted non-assessable terms for potential adverse drug reactions, including pregnancy-related exposure and diagnostic procedure terms from MedDRA, to maintain methodological alignment with analogous research studies. Gestational trimesters were categorized as: first trimester (weeks 0-13), second trimester (weeks 14-27), and third trimester (weeks 28 onward) (Moro et al., 2016). Spontaneous abortion (SAB) referred to fetal loss occurring before 20 weeks of gestation, while stillbirth denoted intrauterine death at or beyond 20 weeks. Preterm birth was characterized by delivery of a live infant prior to 37 completed gestational weeks. Reports fulfilling one or more predefined criteria were included in the final analytical cohort, with rigorous exclusion of non-U.S. submissions and duplicate records through standardized deduplication protocols. In alignment with previously established methodology (Datwani et al., 2015). For the case reports ultimately included in the study, data extraction was performed separately. The extracted data included: vaccine manufacturer, sex, age, number of reported cases, AEs, time of onset, vaccination frequency, outcome, and severity (whether classified as a SAE)).

### 2.3 Statistics methods

Currently, the primary method for mining AE signals globally is disproportionality methods, which can reduce the workload of clinical research and focus its scope effectively (Chen et al., 2023; Shu et al., 2022). In this study, we employed the Reporting Odds Ratio (ROR) and Bayesian Confidence Propagation Neural Network (BCPNN) approaches to systematically identify potential safety signals - defined as Tdap-AE combinations exhibiting disproportionate reporting patterns relative to all other vaccines in the VAERS database. The comparator group for all disproportionality methods consisted of all other non-Tdap vaccine reports from individuals within the VAERS database during the same study period. The analytical framework was built upon standard 2×2 contingency tables (**Supplementary Table S1**), with calculations yielding ROR point (**Supplementary Table S2**) estimates and their associated 95% confidence intervals. This methodology quantifies the relative reporting frequency of specified adverse outcomes following Tdap vaccination during the observation period within the VAERS reporting system. The signal strength, reflected by the magnitude of ROR values, serves as a quantitative indicator of potential vaccine-AE associations. All statistical computations were performed using R statistical software (version 4.4.1) and SPSS (version 19.0; IBM Corp.), with predefined threshold criteria for ROR-based signal detection (detailed in **Supplementary Table S2**). A risk signal was considered statistically significant only when exceeding all established methodological thresholds: Lower limit of 95%CI > 1, the case count≥3. The extremely high ROR value may not reflect a significant risk for rare pregnancy outcomes, but rather be caused by a very low background reporting rate or an increased reporting awareness during a specific period. Therefore, the ROR magnitude itself should not be misconstrued as a direct measure of effect size or clinical impact. The primary utility of these signals is for hypothesis generation.

## 3 Results

### 3.1 Descriptive analysis

**Figure 1:**
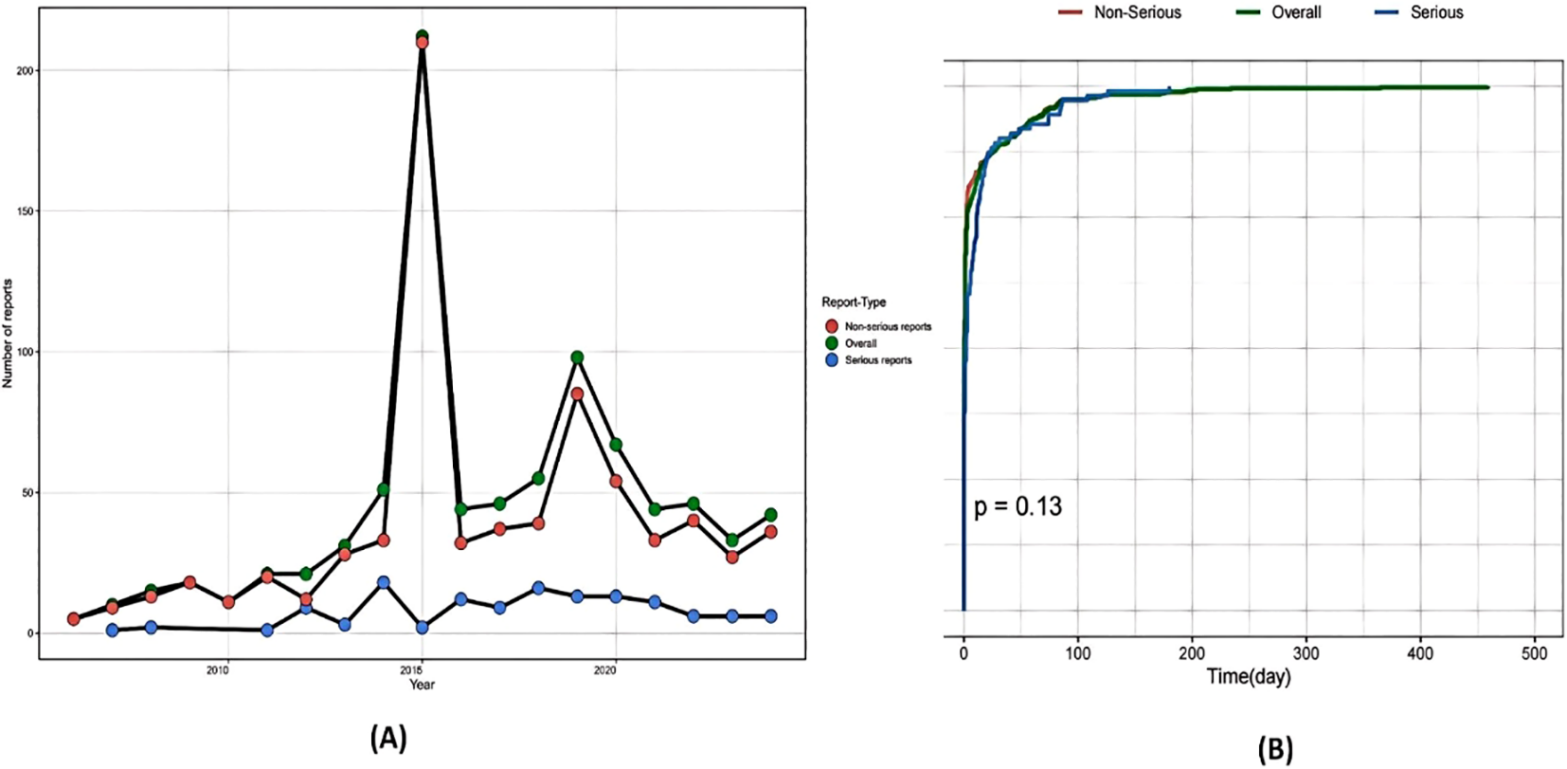
Annual trends in adverse event reports. **(A)** The annual reporting quantities for different report types; **(B)** Survival curve analysis of the occurrence time of adverse events.

During the surveillance period from June 10, 2005 to December 31, 2024, the VAERS documented 20,358 U.S. reports associated with Tdap vaccination use alone. Through automated retrieval coupled with manual validation, 870 pregnancy-related reports were identified as meeting study inclusion criteria. All reports were from female individuals, consistent with our pregnancy focus. The cohort’s age ranged from 15 to 49 years, with a median age of 30 years. Of the pregnancy reports with documented timing of immunization, the vast majority (520; 78.7%) documented receipt of the Tdap vaccine during the final trimester. As detailed in **Table 1**, 128 cases (14.71%) were adjudicated as SAEs according to regulatory definitions. The predominant SAE manifestation was hospitalization/prolonged hospitalization (n=105, 82.0% of SAEs). Temporal analysis revealed that 71.9% of reported AEs occurred within 30 days post-vaccination, with the earliest onset recorded on day 0 (vaccination day). First-trimester vaccination accounted for 62.3% of pregnancy-related AE reports. Brand-specific analysis identified Adacel as the most frequently reported Tdap vaccine (51.7% of cases). Longitudinal surveillance demonstrated peak AE reporting in 2015, followed by a marked decline during the COVID-19 pandemic period (2020-2022), with a subsequent modest resurgence observed since 2023 (**Figure 1**).

**Table 1:** Characteristics of Tdap vaccine reports in pregnant women from VAERS.

Characteristic	Non-serious reports	Serious reports	Overall
Tdap administered alone, N	742	128	870
Maternal age in years, median (range)	30 [15, 49]	31 [20, 46]	30 [15, 49]
Interval from vaccination to adverse event in days			
0–30 days	527	99	626
31–60 days	29	4	33
61–90 days	23	5	28
91–120 days	4	1	5
121–150 days	2	1	3
151–180 days	3	1	4
181–360 days	4	0	4
>360 days	2	0	2
median (range)	0 [0, 459]	2[0, 180]	0 [0, 459]
Trimester of pregnancy at time of vaccination			
First (0–13 wk)	27	33	60
Second (14–27 wk)	45	36	81
Third (≥28 wk)	274	246	520
Dose series			
1	254	34	288
2	47	5	52
3	3	0	3
>3	12	4	16
Unknown	426	85	511
Serious outcome			
Death	0	0	0
Life-threatening	0	18	18
Requires hospitalization or prolongs existing hospitalization	0	105	105
Disability	0	24	24
Brand name of Tdap vaccine, N (%)			
Adacel	381 (51.3)	69 (53.9)	450(51.7)
Boostrix	301 (40.6)	45 (35.2)	346 (39.8)
Unknown	60 (8.1)	14 (10.9)	74 (8.5)

VAERS: Vaccine Adverse Event Reporting System

### 3.2 Disproportionality analysis

**Figure 2:**
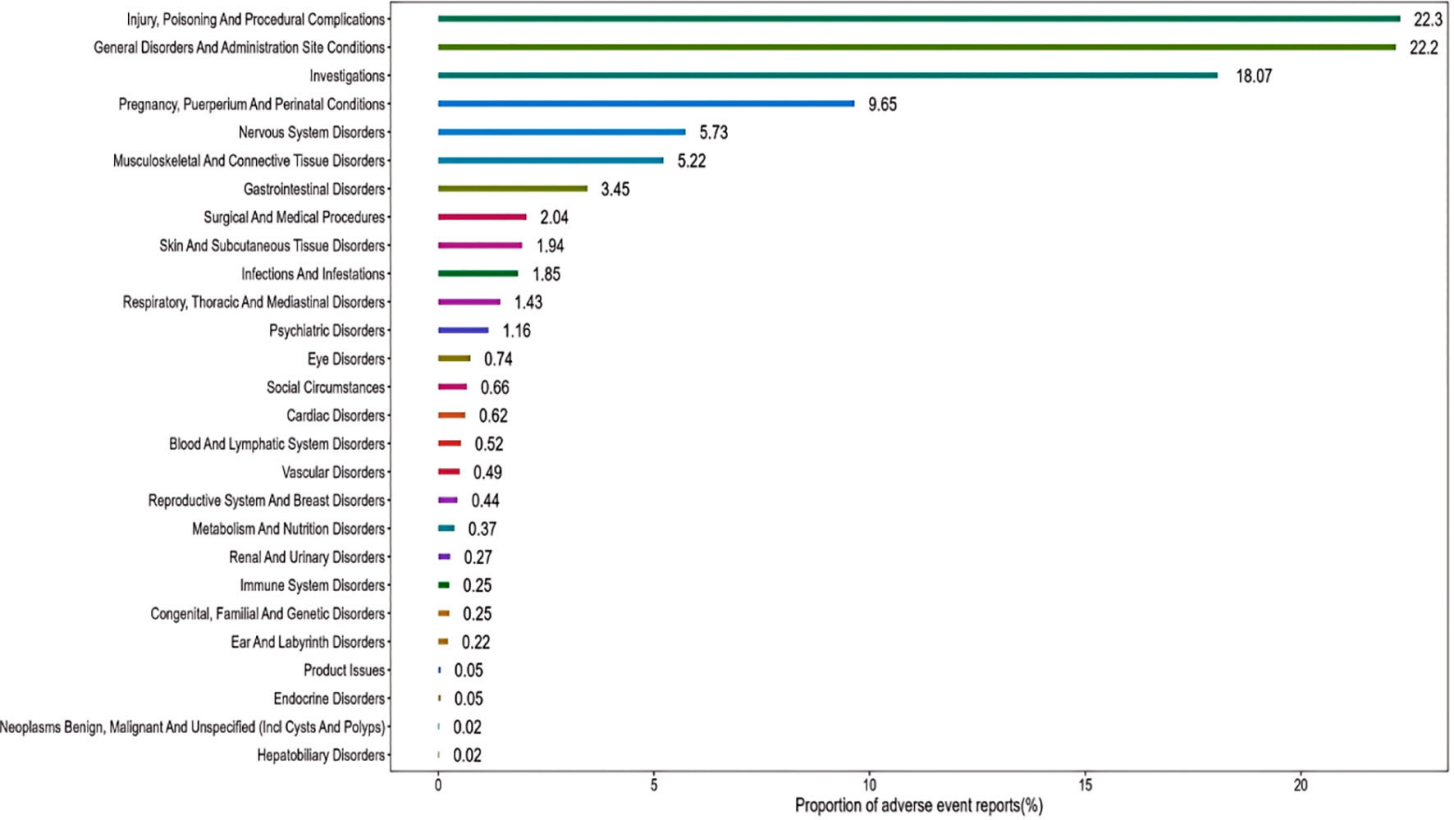
The proportions of various System Organ Classes (SOCs) cases related to Tdap vaccination.

**Figure 3:**
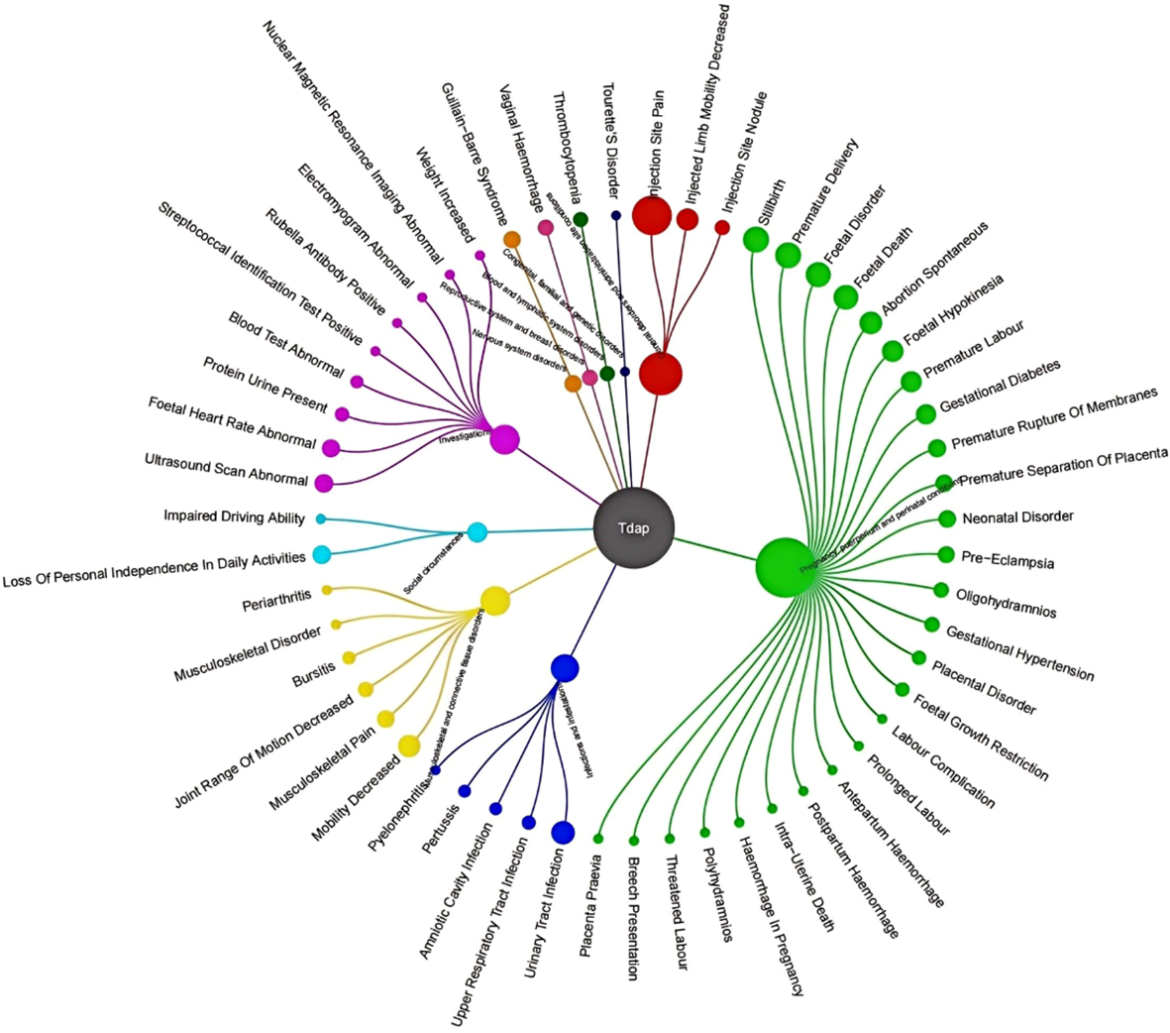
Mapping projection of Tdap-Associated Preferred Terms (PTs) across SOCs. This diagram illustrates the hierarchical relationship and distribution of individual PTs within their respective parent SOCs. The size of the bubble represents the number of cases for each PT. This visualization does not depict a correlation between two continuous variables, but rather a structured mapping of the MedDRA terminology used in this study.

These findings represent potential safety signals from a spontaneous reporting system and should not be interpreted as evidence of causal association or increased population risk. The pharmacovigilance assessment encompassed 4,063 documented Tdap vaccine-adverse event pairs. To ensure analytical rigor, cases associated with improper vaccine storage, routine laboratory abnormalities, or administration errors were systematically excluded as non-relevant confounding factors. Application of the predefined signal detection algorithm yielded 113 preferred term (PT) level signals from the Tdap vaccine safety surveillance data. These signals spanned 27 system organ classes (SOCs), with their proportional distribution illustrated in **Figures 2**, **3**. The three most frequently identified SOC categories were: Injury, poisoning and procedural complications (22.3% of signals); General disorders and administration site conditions (22.2%); Investigations (18.07%).

**Figure 4:**
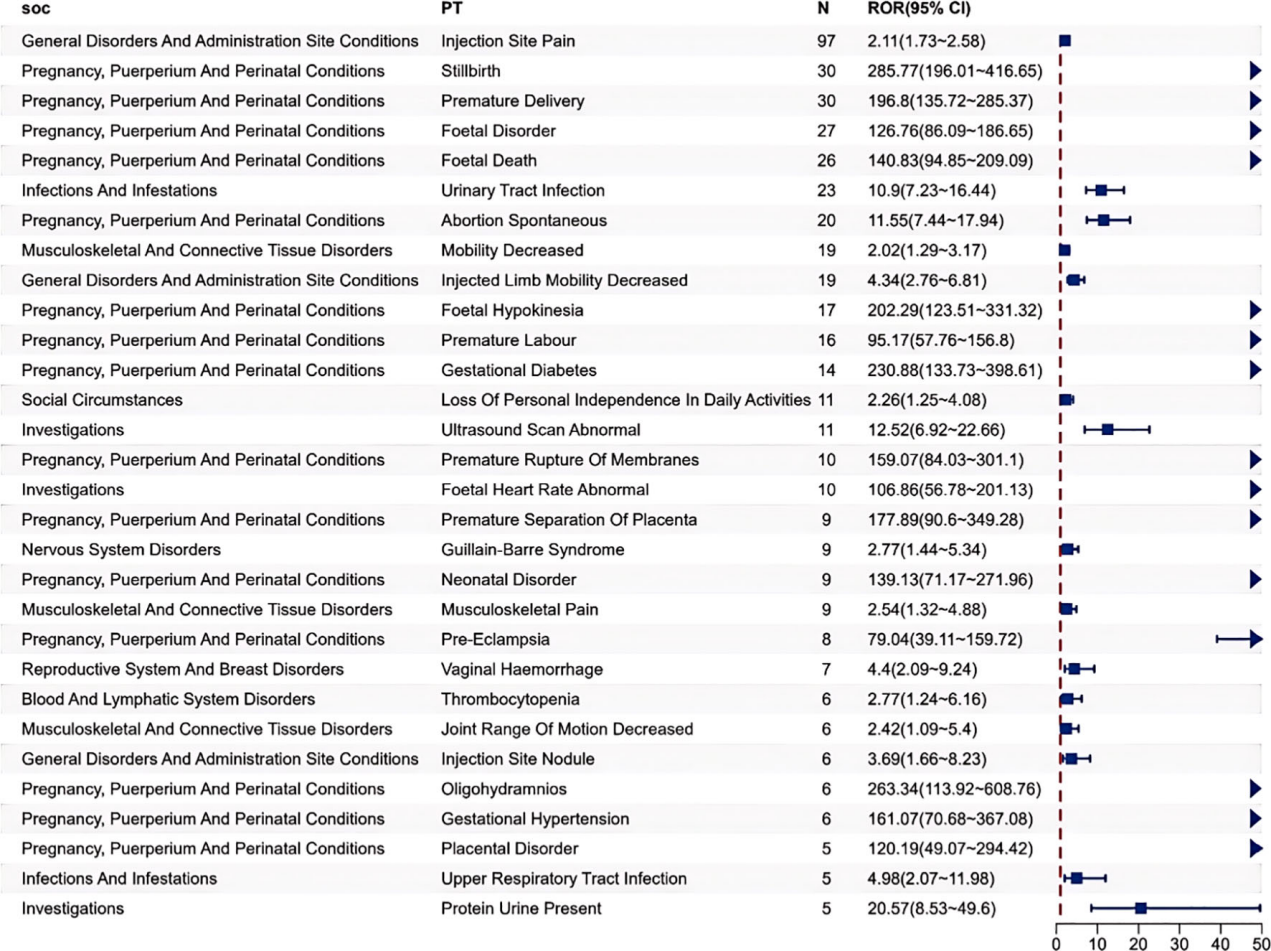
Disproportionality analysis of preferred terms (PT) with top 30 occurrence frequency of Tdap vaccination-related AE signals Only the PT judged as positive signals were presented.

Sort the PTs that meet the threshold in descending order of the number of signals, and select the top 30 PTs (**Supplementary Table S3** and **Figure 4**). Most of the PTs are consistent with the product instructions and previous studies (Moro et al., 2016), which proves the feasibility of this research. The five most frequent Tdap-related AE signals were injection site pain (n=97, ROR 2.11, IC 1.06), stillbirth (n=30, ROR 285.77, IC 8.01), premature delivery (n=30, ROR 196.8, IC 7.51), foetal disorder (n=27, ROR 126.76, IC 6.91), and foetal death (n=26, ROR 140.83, IC 7.06). However, the effective sample size for signal detection of specific outcomes like stillbirth, fetal death, and preterm delivery remains limited by the actual number of these rare events reported. The AE signals not mentioned in the vaccine instructions mainly include SOC of Pregnancy, puerperium and perinatal conditions, such as premature delivery and foetal hypokinesia. Furthermore, there are also adverse reactions from other SOCs, such as urinary tract infection and mobility decreased. The most common pregnancy-specific outcome is stillbirth (n=30, ROR 285.77, IC 8.01) and premature delivery (n=30, ROR 196.8, IC 7.51), and the next one is abortion spontaneous (n=20, ROR 11.55, IC). The most common non-pregnancy-specific outcome is injection site reaction, such as injection site pain (n=97, ROR 2.11, IC 1.06). Meanwhile, there were 9 cases of Guillain-Barre Syndrome among the Nervous system disorders. The 92 reports indicated adverse outcomes for infants or fetuses, including 56 cases of fetal death and 36 cases of fetal diseases.

**Figure 5:**
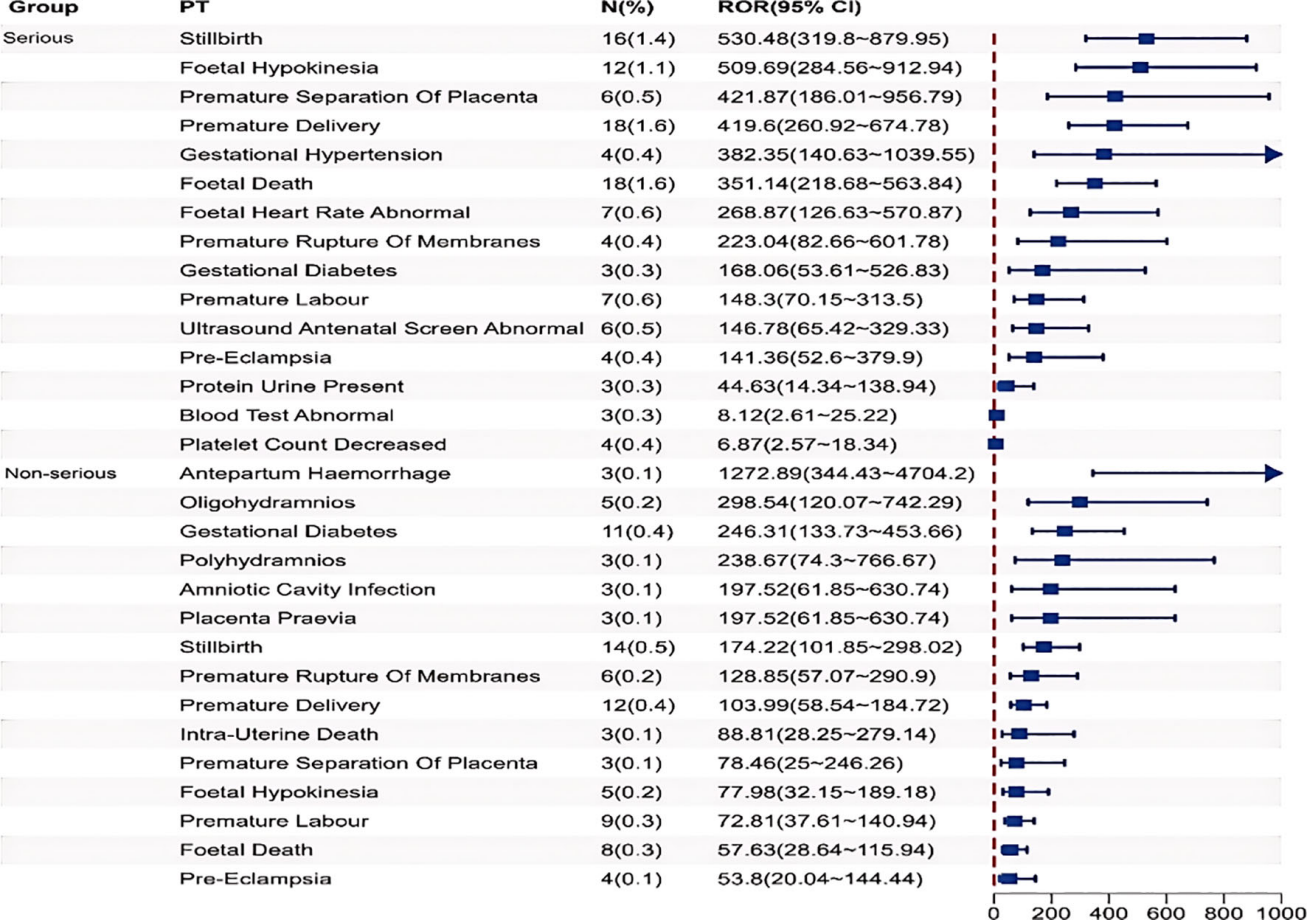
Comparison of the top 15 positive signal intensity of Tdap vaccination-related severe and non-severe AEs.

**Supplementary Table S4** and **Figure 5** demonstrates distinct patterns in the distribution of serious versus non-serious adverse event (AE) signals associated with Tdap vaccination, focusing on the top 15 signals ranked by positive signal intensity. Firstly, the SOC classification for the severe adverse event group includes not only the common conditions such as Pregnancy, Puerperium and Perinatal conditions, but also Investigations (such as AE signals of foetal heart rate abnormal, protein urine present, and platelet count decreased). Secondly, the top 5 signal strengths of AEs in the severe and non-severe event groups are different.

Our pharmacovigilance investigation identified six documented cases of chorioamnionitis temporally associated with Tdap vaccination (**Table 2**). The case distribution demonstrated geographical diversity, with individual reports originating from five distinct U.S. states: California, Florida, Minnesota, New Hampshire, and South Carolina. Furthermore, there was 1 case of a pregnant woman experiencing a serious adverse event, characterized by “Requires hospitalization or prolongs”. The median onset time of chorioamnionitis reports was 7 days. Most pregnant women (50.0%) receive the Tdap vaccination during the third trimester. 66.7% of the reports identified at least one risk factor for chorioamnionitis.

**Table 2:** Characteristics of chorioamnionitis reports (N = 6) in the VAERS.

Characteristic	Case number and case proportion
State	
California	1 (16.7%)
Florida	1 (16.7%)
Minnesota	1 (16.7%)
New Hampshire	1 (16.7%)
South Carolina	1 (16.7%)
Unknown	1 (16.7%)
Age (year)	
< 18	1 (16.7%)
18-35	5 (83.3%)
Outcome	
Non-Serious	5 (83.3%)
Serious	1 (16.7%)
Serious outcome	
Requires hospitalization or prolongs	1
Interval from vaccination to adverse event in days	
Median	7
Trimester of vaccination	
First (0–13 weeks)	1 (16.7%)
Second (14–27 weeks)	1 (16.7%)
Third (≥28 weeks)	3 (50.0%)
Unknown	1 (16.7%)
Risk factors for Chorioamnionitis	
No risk factors identified	2 (33.3%)
At least one risk factor identified	4 (66.7%)
Nulliparity	1
Preterm premature rupture of membranes	2
Prolonged labor	1
Lower genitourinary infections	2
Internal fetal monitoring	0
Brand name of Tdap vaccine	
Adacel	3 (50.0%)
Boostrix	3 (50.0%)

## 4 Discussion

Infants under 3 months of age are highly susceptible to pertussis infection and are prone to life-threatening complications (such as pneumonia, epilepsy, brain damage, etc.). Vaccination with Tdap during pregnancy is the most effective method for preventing pertussis infection in infants and young children. Some countries recommend vaccinating during pregnancy with the Tdap vaccine, but the vaccination rates vary. In 2014, the vaccination rate in the UK was 60%, while in 2013-2014, it was 50% in the US (Koepke et al., 2015). The main reason for the low vaccination rate is the concerns of clinicians or women regarding the safety of vaccinating during pregnancy with the TdaP vaccine (Chamberlain et al., 2015). This large-scale pharmacoepidemiological study provides comprehensive insights into the safety profile of Tdap vaccination during pregnancy through systematic analysis of VAERS data (2005-2024). As a spontaneous reporting system, VAERS provides clinically relevant safety data on vaccines under real-world conditions. While it serves as an important tool for detecting potential safety signals, it cannot establish causal relationships between vaccination and adverse events. Instead, its key function is to identify possible risks requiring additional scientific evaluation. While current guidelines recommend Tdap vaccination in late second or third trimester, reports of first-trimester vaccination in VAERS may stem from: 1) immunization before pregnancy recognition; 2) vaccination prior to confirmed pregnancy status; and 3) occasional clinical decisions for early vaccination, reflecting real-world practice patterns. Overall, the AEs reported were consistent with those observed in the pre-market trials of the vaccine and the studies conducted after its early approval, including injection site reactions (pain, hardening) and systemic reactions (Moro et al., 2016). Our findings corroborate existing evidence while identifying novel risk signals that warrant careful consideration in clinical practice and pharmacovigilance systems.

The adverse event (AE) profile revealed significant differences between pregnancy-specific and non-pregnancy-specific events. Injection site pain (ROR 2.11, IC 1.06) was consistent with the established safety profile of aluminum-adjuvanted vaccines and product labeling information. The predominance of general disorders and administration site conditions (22.2% of all SOC reports) reflects expected immune activation responses. Extensive clinical trial data and post-marketing surveillance reports collectively support the safety of Tdap vaccination during pregnancy (Halperin et al., 2018). Currently, at least 16 clinical trials involving approximately 150,000 pregnant women have evaluated Tdap vaccination during gestation, with individual study sample sizes ranging from 33,000 to 53,000 participants (Maertens et al., 2016a; Moro et al., 2016; Maertens et al., 2016b). These studies evaluated and confirmed the safety of various combined vaccines containing pertussis antigen components used in the global immunization program.

As expected, the most common non-pregnancy-specific outcome was injection site reaction, accounting for 17.4%; in the pre-approval trials conducted in non-pregnant populations, injection site reaction has been identified as a common adverse event (Zheteyeva et al., 2012). Spontaneous abortion represented the predominant pregnancy-specific outcome, documented in 18.8% of case reports. This event occurs naturally in approximately 15-20% of gestations (Zheteyeva et al., 2012).

Our disproportionality assessment, evaluating the observed-to-expected reporting ratios for specific vaccine-adverse event combinations within VAERS, identified statistically significant signals for stillbirth (ROR 285.77, IC 8.01) and fetal death (ROR 140.83, IC 7.06). These findings suggest a higher-than-anticipated reporting frequency following Tdap vaccination compared to the background reporting distribution in the database. However, due to the spontaneous reporting nature of VAERS and its methodological constraints, these results cannot substantiate a causal relationship between Tdap immunization and the reported pregnancy outcomes. Strong signals for stillbirth and fetal death require further mechanistic evaluation. Stillbirths were evaluated in one randomized controlled trial and four observational studies, none of which identified an elevated risk associated with Tdap vaccination during pregnancy (Vygen-Bonnet et al., 2020). Meanwhile, neonatal mortality was assessed in two observational studies, with sporadic cases reported but no statistically significant correlation to antenatal Tdap immunization identified (Donegan et al., 2014; Morgan et al., 2015). We speculate that this is related to the recommendation of administering the vaccine during the late stage of pregnancy. While temporal association exists (median onset: 7 days for chorioamnionitis), 66.7% of cases had identifiable obstetric risk factors (e.g., preterm rupture of membranes), suggesting potential confounding.

Chorioamnionitis, a polymicrobial inflammatory condition affecting fetal membranes, amniotic cavity/fluid, and placenta, typically results from ascending bacterial infection (Oh et al., 2017). It can manifest at any gestational stage or during delivery, often associated with premature membrane rupture. Diagnosis relies on clinical, microbiological, and/or histopathological criteria. This clinically significant condition frequently precipitates preterm labor and may progress to neonatal sepsis (Vygen-Bonnet et al., 2020). Six observational studies examined chorioamnionitis risk, consistently demonstrating elevated incidence among pregnant individuals receiving Tdap vaccination (Vygen-Bonnet et al., 2020). In our study, only six reports of choriamnionitis were identified over approximately 20 years of surveillance, with most cases having documented obstetric risk factors. This is also consistent with a study covering the entire VAERS database over a period of 24 years, which found that any vaccine rarely reported chorioamnionitis AE (Datwani et al., 2015). According to previous reports, in the VAERS reports, nearly 60% of women with chorioamnionitis had at least one risk factor for this condition (Datwani et al., 2015). Similarly, in this study, 66.7% of the reports identified at least one risk factor for chorioamnionitis. Therefore, this signal is better interpreted as illustrative of background risk and potential confounding rather than a primary study finding. Establishing a biological mechanism linking vaccination to chorioamnionitis remains challenging. Current hypotheses suggest inflammatory pathways may mediate certain adverse pregnancy outcomes, including gestational hypertension and preeclampsia (Datwani et al., 2015). Immunologically, vaccination may plausibly induce gestational inflammatory responses. Pregnancy involves dynamic immune adaptations: from first-trimester localized inflammation during fetal implantation, through mid-gestational immune tolerance, to third-trimester pro-inflammatory signaling initiating parturition (Mor et al., 2017). Tdap immunization represents just one of numerous immune-activating exposures during gestation. Currently, no published animal studies have specifically investigated vaccine-induced immune stimulation and its potential impact on pregnancy outcomes.

Our research benefits from utilizing VAERS, an extensive post-marketing surveillance platform that provides nationwide coverage, enables near real-time safety monitoring, and demonstrates particular sensitivity in identifying uncommon adverse events following immunization. This study restricted its analysis to reports where Tdap was administered alone. While this enhances the specificity of the signal by reducing confounding from co-administered antigens, it may also limit the direct generalizability of the findings to real-world clinical practice. In addition, VAERS has the inherent limitations of all passive monitoring systems, including underreporting, reporting bias, and inconsistent reporting quality. Therefore, the VAERS data must be interpreted with caution, as it is generally not suitable for assessing causal relationships. One significant limitation of VAERS is that it is unable to calculate the incidence or prevalence of adverse events, as no data on the number of pregnant women who received the vaccine has been collected. The representative of incidence or prevalence is the crude reporting rate, and if the data on the number of Tdap doses used or distributed by pregnant women is known, the crude reporting rate can be calculated. However, the data on Tdap vaccination coverage during pregnancy is limited, so it is difficult to estimate the reporting rate of pregnancy-related diseases using VAERS.

## 5 Conclusion

This large-scale pharmacovigilance study confirms Tdap’s fsafety signals for maternal immunization from a passive surveillance system. While novel or strong signals warrant investigation, the low absolute risk supports maintained recommendations while guiding enhanced monitoring strategies for high-risk pregnancies. These results should not be interpreted as evidence of causal association or increased population risk, particularly those based on limited reports, which required further validation in active surveillance studies.

## Data Availability

The original contributions presented in the study are included in the article/**Supplementary Material**. Further inquiries can be directed to the corresponding author/s.
