# Supplementary material for: Risk signal assessment of Tdap vaccine use alone by pregnant women: An updated pharmacoepidemiological study
Source: Front Cell Infect Microbiol. 2026 Jan 27;15:1689560. doi: 10.3389/fcimb.2025.1689560 (PMC12886459; doi:10.3389/fcimb.2025.1689560)
Supplement: Supplementary file 4 [file Table1.docx]

**Table S1.** Four-grid Table of disproportionality analysis.

|  | Target AE | Other AEs | Total |
| --- | --- | --- | --- |
| Tdap vaccine | a | b | a+b |
| Other vaccines | c | d | c+d |
| Total | a+c | b+d | N=a+b+c+d |

AE:adverse events; Tdap: tetanus-diphtheria-acellular pertussis

**Table S2.** Disproportionality analysis used for signal detection.

| Method | Calculation formulas | Criteria |
| --- | --- | --- |
| ROR | ROR＝(a/c)/(b/d) | 95%CI > 1, a≥3 |
|  | 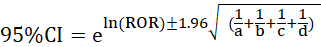 |  |
| BCPNN | IC=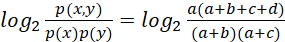 | IC-2SD*>0* |
|  | IC-2SD=E(IC)-2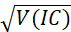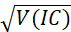 |  |

ROR: Reporting Odds Ratio ; BCPNN:Bayesian confidence propagation neural network

**Table S3.** Disproportionality analysis of preferred terms (PT) with top 30 occurrence frequency of Tdap vaccination-related AE signals

| PT | SOC | N | ROR(95%CI) | IC(IC-2SD) |
| --- | --- | --- | --- | --- |
| Injection Site Pain | General disorders and administration site conditions | 97 | 2.11(1.73-2.58) | 1.06(0.77) |
| Stillbirth | Pregnancy, puerperium and perinatal conditions | 30 | 285.77(196.01-416.65) | 8.01(7.46) |
| Premature Delivery | Pregnancy, puerperium and perinatal conditions | 30 | 196.8(135.72-285.37) | 7.51(6.97) |
| Foetal Disorder | Pregnancy, puerperium and perinatal conditions | 27 | 126.76(86.09-186.65) | 6.91(6.35) |
| Foetal Death | Pregnancy, puerperium and perinatal conditions | 26 | 140.83(94.85-209.09) | 7.06(6.49) |
| Urinary Tract Infection | Infections and infestations | 23 | 10.9(7.23-16.44) | 3.43(2.84) |
| Abortion Spontaneous | Pregnancy, puerperium and perinatal conditions | 20 | 11.55(7.44-17.94) | 3.52(2.89) |
| Mobility Decreased | Musculoskeletal and connective tissue disorders | 19 | 2.02(1.29-3.17) | 1.01(0.36) |
| Injected Limb Mobility Decreased | General disorders and administration site conditions | 19 | 4.34(2.76-6.81) | 2.11(1.46) |
| Foetal Hypokinesia | Pregnancy, puerperium and perinatal conditions | 17 | 202.29(123.51-331.32) | 7.55(6.85) |
| Premature Labour | Pregnancy, puerperium and perinatal conditions | 16 | 95.17(57.76-156.8) | 6.52(5.81) |
| Gestational Diabetes | Pregnancy, puerperium and perinatal conditions | 14 | 230.88(133.73-398.61) | 7.73(6.95) |
| Loss Of Personal Independence In Daily Activities | Social circumstances | 11 | 2.26(1.25-4.08) | 1.17(0.34) |
| Ultrasound Scan Abnormal | Investigations | 11 | 12.52(6.92-22.66) | 3.64(2.8) |
| Premature Rupture Of Membranes | Pregnancy, puerperium and perinatal conditions | 10 | 159.07(84.03-301.1) | 7.23(6.33) |
| Foetal Heart Rate Abnormal | Investigations | 10 | 106.86(56.78-201.13) | 6.68(5.79) |
| Premature Separation Of Placenta | Pregnancy, puerperium and perinatal conditions | 9 | 177.89(90.6-349.28) | 7.38(6.44) |
| Guillain-Barre Syndrome | Nervous system disorders | 9 | 2.77(1.44-5.34) | 1.47(0.55) |
| Neonatal Disorder | Pregnancy, puerperium and perinatal conditions | 9 | 139.13(71.17-271.96) | 7.05(6.11) |
| Musculoskeletal Pain | Musculoskeletal and connective tissue disorders | 9 | 2.54(1.32-4.88) | 1.34(0.43) |
| Pre-Eclampsia | Pregnancy, puerperium and perinatal conditions | 8 | 79.04(39.11-159.72) | 6.26(5.28) |
| Vaginal Haemorrhage | Reproductive system and breast disorders | 7 | 4.4(2.09-9.24) | 2.13(1.11) |
| Thrombocytopenia | Blood and lymphatic system disorders | 6 | 2.77(1.24-6.16) | 1.47(0.37) |
| Joint Range Of Motion Decreased | Musculoskeletal and connective tissue disorders | 6 | 2.42(1.09-5.4) | 1.27(0.18) |
| Injection Site Nodule | General disorders and administration site conditions | 6 | 3.69(1.66-8.23) | 1.88(0.79) |
| Oligohydramnios | Pregnancy, puerperium and perinatal conditions | 6 | 263.34(113.92-608.76) | 7.91(6.76) |
| Gestational Hypertension | Pregnancy, puerperium and perinatal conditions | 6 | 161.07(70.68-367.08) | 7.25(6.12) |
| Placental Disorder | Pregnancy, puerperium and perinatal conditions | 5 | 120.19(49.07-294.42) | 6.85(5.64) |
| Upper Respiratory Tract Infection | Infections and infestations | 5 | 4.98(2.07-11.98) | 2.31(1.13) |
| Protein Urine Present | Investigations | 5 | 20.57(8.53-49.6) | 4.35(3.17) |

PT: Preferred Terms; SOC: system organ class

**Table S4.** Comparison of the top 15 positive signal intensity of Tdap vaccination-related severe and non-severe AEs

| PT | SOC | N | ROR(95%CI) | IC(IC-2SD) |
| --- | --- | --- | --- | --- |
| **Serious** |  |  |  |  |
| Stillbirth | Pregnancy, puerperium and perinatal conditions | 16 | 530.48(319.8-879.95) | 8.96(8.23) |
| Foetal Hypokinesia | Pregnancy, puerperium and perinatal conditions | 12 | 509.69(284.56-912.94) | 8.91(8.08) |
| Premature Separation Of Placenta | Pregnancy, puerperium and perinatal conditions | 6 | 421.87(186.01-956.79) | 8.65(7.53) |
| Premature Delivery | Pregnancy, puerperium and perinatal conditions | 18 | 419.6(260.92-674.78) | 8.63(7.95) |
| Gestational Hypertension | Pregnancy, puerperium and perinatal conditions | 4 | 382.35(140.63-1039.55) | 8.52(7.2) |
| Foetal Death | Pregnancy, puerperium and perinatal conditions | 18 | 351.14(218.68-563.84) | 8.38(7.7) |
| Foetal Heart Rate Abnormal | Investigations | 7 | 268.87(126.63-570.87) | 8.02(6.99) |
| Premature Rupture Of Membranes | Pregnancy, puerperium and perinatal conditions | 4 | 223.04(82.66-601.78) | 7.76(6.45) |
| Gestational Diabetes | Pregnancy, puerperium and perinatal conditions | 3 | 168.06(53.61-526.83) | 7.37(5.9) |
| Premature Labour | Pregnancy, puerperium and perinatal conditions | 7 | 148.3(70.15-313.5) | 7.18(6.15) |
| Ultrasound Antenatal Screen Abnormal | Investigations | 6 | 146.78(65.42-329.33) | 7.17(6.07) |
| Pre-Eclampsia | Pregnancy, puerperium and perinatal conditions | 4 | 141.36(52.6-379.9) | 7.12(5.81) |
| Protein Urine Present | Investigations | 3 | 44.63(14.34-138.94) | 5.47(4.02) |
| Blood Test Abnormal | Investigations | 3 | 8.12(2.61-25.22) | 3.02(1.57) |
| Platelet Count Decreased | Investigations | 4 | 6.87(2.57-18.34) | 2.77(1.48) |
| **Non-serious** |  |  |  |  |
| Antepartum Haemorrhage | Pregnancy, puerperium and perinatal conditions | 3 | 1272.89(344.43-4704.2) | 9.9(8.25) |
| Oligohydramnios | Pregnancy, puerperium and perinatal conditions | 5 | 298.54(120.07-742.29) | 8.11(6.88) |
| Gestational Diabetes | Pregnancy, puerperium and perinatal conditions | 11 | 246.31(133.73-453.66) | 7.85(6.99) |
| Polyhydramnios | Pregnancy, puerperium and perinatal conditions | 3 | 238.67(74.3-766.67) | 7.81(6.31) |
| Amniotic Cavity Infection | Infections and infestations | 3 | 197.52(61.85-630.74) | 7.55(6.06) |
| Placenta Praevia | Pregnancy, puerperium and perinatal conditions | 3 | 197.52(61.85-630.74) | 7.55(6.06) |
| Stillbirth | Pregnancy, puerperium and perinatal conditions | 14 | 174.22(101.85-298.02) | 7.37(6.61) |
| Premature Rupture Of Membranes | Pregnancy, puerperium and perinatal conditions | 6 | 128.85(57.07-290.9) | 6.96(5.85) |
| Premature Delivery | Pregnancy, puerperium and perinatal conditions | 12 | 103.99(58.54-184.72) | 6.66(5.84) |
| Intra-Uterine Death | Pregnancy, puerperium and perinatal conditions | 3 | 88.81(28.25-279.14) | 6.44(4.97) |
| Premature Separation Of Placenta | Pregnancy, puerperium and perinatal conditions | 3 | 78.46(25-246.26) | 6.26(4.8) |
| Foetal Hypokinesia | Pregnancy, puerperium and perinatal conditions | 5 | 77.98(32.15-189.18) | 6.25(5.06) |
| Premature Labour | Pregnancy, puerperium and perinatal conditions | 9 | 72.81(37.61-140.94) | 6.15(5.23) |
| Foetal Death | Pregnancy, puerperium and perinatal conditions | 8 | 57.63(28.64-115.94) | 5.82(4.85) |
| Pre-Eclampsia | Pregnancy, puerperium and perinatal conditions | 4 | 53.8(20.04-144.44) | 5.73(4.43) |


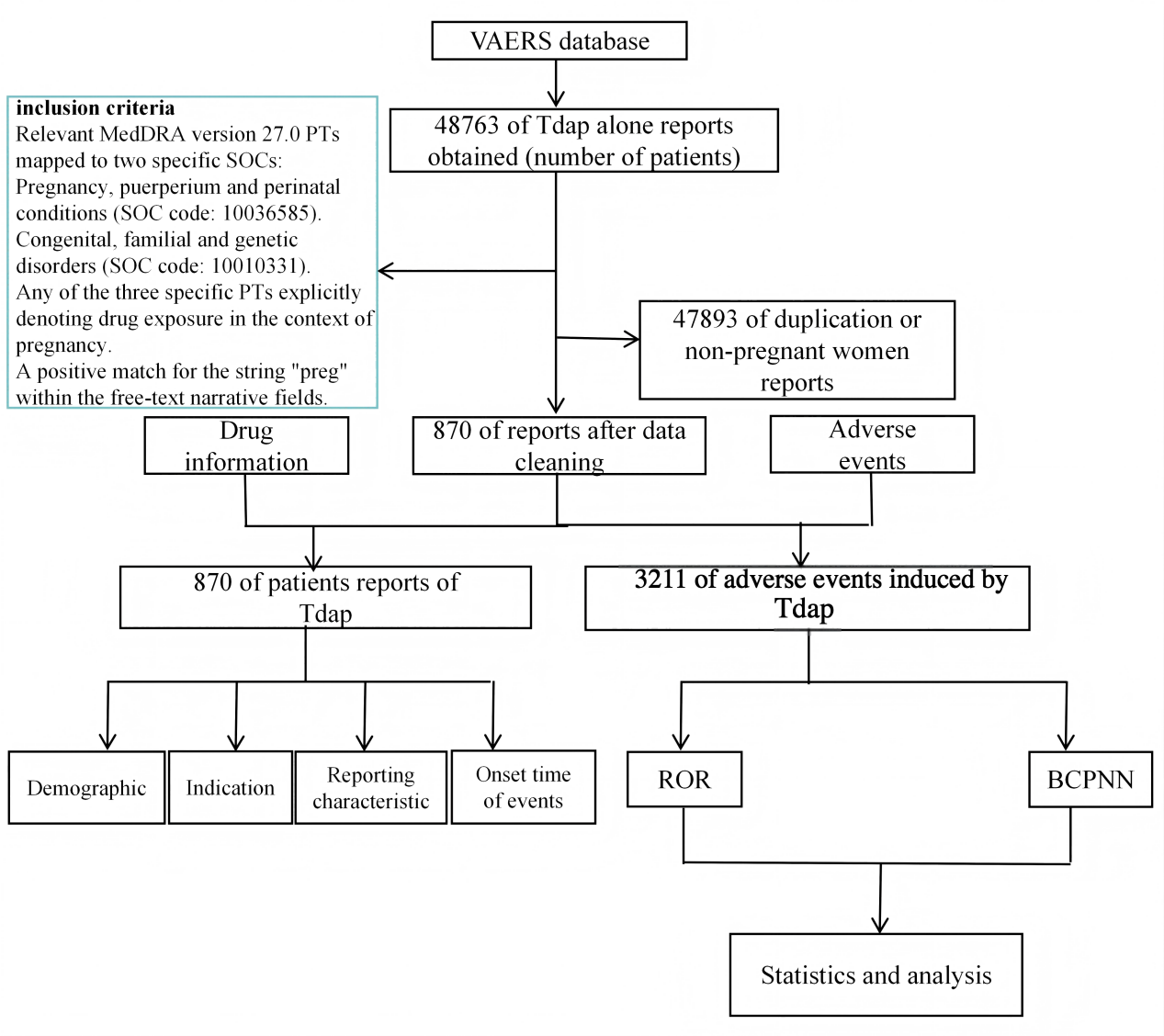


**Figure S1.** Flow diagram for the selection of adverse events (AE) associated with Tdap from VAERS database

VAERS: Vaccine Adverse Event Reporting System; Tdap: tetanus toxoid, reduced diphtheria toxoid, and acellular pertussis vaccine; MedDRA: the Medical Dictionary for Regulatory Activities; PT: Preferred Terms; SOC: system organ class; ROR: Reporting Odds Ratio ; BCPNN: Bayesian confidence propagation neural network
